# Effects of Exercise and Physical Activity Levels on Childhood Cancer: An Umbrella Review

**DOI:** 10.3390/healthcare11060820

**Published:** 2023-03-10

**Authors:** Christina Rapti, Petros C. Dinas, Costas Chryssanthopoulos, Alexandra Mila, Anastassios Philippou

**Affiliations:** 1 Department of Physiology, Medical School, National and Kapodistrian University of Athens, 11527 Athens, Greece; 2 FAME Laboratory, Department of Physical Education and Sport Science, University of Thessaly, 42100 Trikala, Greece

**Keywords:** malignancy, physical training, children, adolescents, systematic review

## Abstract

Patients and survivors of childhood cancer experience adverse effects related to the disease and its treatment. These adverse effects are associated with both physiological and psychological health. Exercise helps manage the side effects and improve the health outcomes. The objective of this umbrella review is to search the current literature in the context of exercise and physical activity as complementary interventions on pediatric cancer and to provide comprehensive information about the derived health outcomes. A literature search was conducted on the Cochrane, PubMed, and Embase databases for systematic reviews published up to January 2023. Moreover, a hand search of reference lists was performed. We included participants under 19 years of age at diagnosis of any type of childhood cancer, without restriction on the type or phase of treatment, who participated in exercise interventions. The results showed a beneficial impact on fatigue, muscle strength, aerobic capacity, activity and participation levels, psychosocial health, cardiovascular/cardiorespiratory fitness, physical function, bone mineral density, and brain volume and structure, with limited and not serious adverse effects. These findings documented that exercise interventions had a positive effect on many physiological and psychological health outcomes in pediatric cancer patients and survivors.

## 1. Introduction

Cancer is a chronic disease that occurs in people of all ages. It can affect any part of the body, and it is divided into two main categories: the hematologic (blood) cancers and the solid tumor cancers [1,2,3]. Cancer begins with genetic changes in single cells, which then grow into a mass (or tumor). This mass can invade other parts of the body and put the life of the individual at risk if left untreated [3].

A cancer diagnosis is upsetting at any age, but especially in younger patients. According to the World Health Organization [3], approximately 400.000 children and adolescents of 0–19 years old develop cancer every year. The most common types of cancer in children and adolescents are leukemias, brain cancers, lymphomas and solid tumors, such as neuroblastoma and Wilms tumors [3]. More specifically, between the ages of birth to 14 years of age, cancer is the second most common cause of death with leukemia being the most common type of childhood cancer (28% of all cancers) [1]. From 15 to 19 years of age, the most common types of cancer are brain and other nervous system tumors (21% of cancers) [1]. Unfortunately, the causes of childhood cancer are not yet well-defined [4].

Pediatric cancer patients and survivors frequently experience adverse health effects related to the disease and its treatment, such as nausea, extreme fatigue, impaired aerobic and anaerobic fitness, lower muscular strength, impaired neuromuscular coordination, impaired pulmonary and physical function, cardiovascular problems, decreased quality of life, pain, muscle mass loss, and reduced energy, among others [2,5,6,7,8,9,10]. Many of the adverse effects persist for years after the end of treatment [9]. The side effects and duration of hospitalization also impact a patient’s ability and willingness to be physically active during treatment [2]. Exercise and physical activity in general are considered safe and can be used as a beneficial complementary therapy for attenuating adverse effects, even during the most aggressive phases of treatment [2,9].

Although many systematic reviews examine the effect of exercise and physical activity interventions on pediatric cancer, there is no clear picture of the physiological and psychological outcomes induced by exercise and physical activity in these cancer patients and survivors. Furthermore, although attempts to summarize the evidence from multiple systematic reviews and research syntheses have been made on the adult cancer population in the form of an umbrella review [11], to the best of the authors’ knowledge, no such attempt has been made for pediatric cancer. Such an umbrella review would offer a rapid review of the available evidence, compare results of systematic reviews, demonstrate consistency or contradiction of findings, but also provide both quantitative and qualitative types of evidence [12].

The primary objective of the current umbrella review was to examine the effects of exercise/physical activity on fatigue, health-related quality of life (HRQOL), pain, cardiovascular/cardiorespiratory fitness (CRF), and physical function on pediatric cancer patients. A secondary objective was to investigate the effects of exercise/physical activity on psychosocial health indices, cognitive function, activity/participation levels, body weight/composition and brain volume/structure on pediatric cancer patients. The derived conclusions from this umbrella review can formulate implications for practice and future research.

## 2. Materials and Methods

The current umbrella review was conducted according to the Cochrane Handbook (Chapter V: Overviews of Reviews) and according to the Preferred Reporting Items for Systematic Reviews and Meta-analyses (PRISMA) guidelines [13]. The current umbrella review was registered with the International Platform of Registered Systematic Review and Meta-analysis Protocols (INPLASY-202270035. https://doi.org/10.37766/inplasy2022.7.0035 (accessed on 7 July 2022)).

### 2.1. Selection Criteria

#### 2.1.1. Types of Studies

We included systematic reviews that examined the effects of exercise or physical activity levels on health outcomes during or after childhood cancer. There was no restriction on whether a control group was included or not.

#### 2.1.2. Types of Participants

We included participants under 19 years of age at diagnosis of any type of childhood cancer and without restriction on the type or phase of treatment. The systematic reviews with both children and adults with cancer were included only when the results of the childhood and adult study populations were reported separately.

#### 2.1.3. Types of Interventions

We included systematic reviews with and without meta-analysis that contained interventions of resistance training, aerobic training, or a combination of them, flexibility, functional, balance and motor skill training, physiotherapy practice sessions, yoga interventions and physical activity games.

The exercise or physical activity interventions could be implemented as additional care during cancer treatment on the maintenance or intense phase of disease. It could also be offered after the participants have entered the survival stage as a form of rehabilitation.

There was no restriction on the location or setting where the interventions took place. That could be the hospital, the physical therapy center, the patient’s home, or anywhere else. Moreover, the duration of the interventions could differ per protocol.

#### 2.1.4. Types of Outcome Measures

We included the following primary outcomes: effects of exercise/physical activity on fatigue, health-related quality of life (HRQOL), pain, cardiovascular/cardiorespiratory fitness (CRF), and physical function (incorporating the terms physical fitness, flexibility, coordination, muscle strength, functional capacity, and physical capacity).

As secondary outcomes, we considered psychosocial health indices, cognitive function, activity/participation levels (incorporating the terms energy consumption and physical activity), body weight and composition (incorporating the terms body mass index-(BMI) and bone mineral density), and brain volume and structure.

Moreover, we assessed any adverse effects that arose from the exercise or physical activity interventions.

### 2.2. Search Methods for Identification of Reviews

#### Electronic Searches and Other Resources

Two independent investigators, CR and PCD, performed a comprehensive algorithmic search on Cochrane, PubMed, and Embase databases for systematic reviews published up to 31 December 2022. Furthermore, we reviewed the reference lists of the eligible systematic reviews to identify additional systematic reviews that had not appeared in the initial search outcome. The key words and algorithm used in the searching procedure can be found in Supplement I (pp. 3–15). All obtained systematic reviews stored in EndNote X9 for further handling.

### 2.3. Data Collection and Analysis

#### 2.3.1. Selection of Reviews

After the duplicates were removed, two authors, CR and CC, identified studies meeting the inclusion criteria. Eligible systematic reviews were those that included: (1) children <19 years old (or older participants with cancer if the initiation of disease was before they reach 19 years) (2) with any type of cancer, without restriction on the (3) type or (4) phase of treatment (they could also be childhood cancer survivors), who followed (5) exercise/physical activity interventions. Disagreements were resolved through discussions with a third author (PCD), who acted as a referee.

#### 2.3.2. Data Extraction and Management

Two authors, CR and AP, performed the data extraction from the eligible publications. The data that were included in the final data extraction table were: (a) year of publication, (b) first authors’ name, (c) number and design of studies that are included in the systematic review, (d) the population characteristics (i.e., the participants’ age, type, and stage of cancer), (e) the intervention characteristics (i.e., the type, the duration, the frequency, the intensity of the exercise interventions, and the settings where the interventions took place), and (f) the outcome measures data (i.e., narratively reported study-level data and/or meta-analyzed data).

The outcome measure data contained anything that was related to the participants’ general and psychosocial health, physical, cardiorespiratory, and cardiovascular fitness, and quality of life. Moreover, all the information reported on adverse effects during the intervention period in the included systematic reviews was collected. A priori data extraction validation was performed between the two authors to ensure concordance between them during the process.

#### 2.3.3. Quality of Evidence

Two investigators, CR and PCD, independently assessed the methodological quality of the eligible systematic reviews using the Assessment of Multiple Systematic Reviews (AMSTAR-2) rating scale. We rated the included reviews as “high”, “moderate”, “low,” or “critically low” overall confidence according to seven critical domains that can affect the validity of a systematic review [14]. We also collected the overall risk of bias of the original studies included in the eligible systematic reviews. These data are reported in Supplement I (Table S1).

#### 2.3.4. Data Synthesis

We used a narrative data synthesis approach; we summarized the outcome data that we have collected from the eligible systematic reviews. We did not directly compare different interventions that have been examined in the different eligible systematic reviews with the intent to determine which intervention is the best or the safest. Instead, we synthesized the data to provide the positive and/or adverse effects on cancer in childhood.

## 3. Results

### 3.1. Description of Included Reviews

#### 3.1.1. Search Outcomes

**Figure 1:**
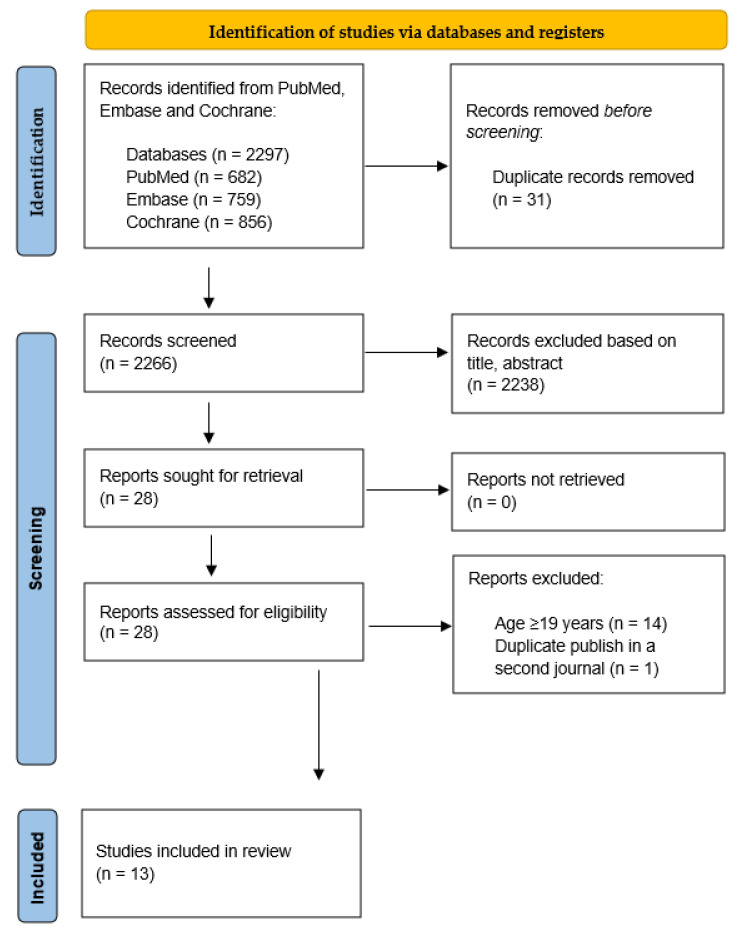
Prisma Flow Chart of the Umbrella Review.

For the umbrella review, we ran the algorithm in the Cochrane Library, PubMed, and Embase electronic databases in December 2021, and again in December 2022. This search revealed 2297 references. After the removal of duplicates, the search resulted in 2266 potentially relevant articles. The screening of titles and abstracts excluded a further 2238 references that did not meet the criteria for inclusion. The 28 remaining references were read in full text. Fourteen systematic reviews did not meet the eligibility criteria of age and were thus excluded. One more systematic review was removed because it was duplicated in a second journal. Therefore, the total number of systematic reviews included in this umbrella review is 13 [5,6,8,15,16,17,18,19,20,21,22,23,24] (PRISMA flow diagram, Figure 1). The number of studies and participants included in the 13 eligible systematic reviews is 145 and 3914, respectively.

#### 3.1.2. Characteristics of Eligible Systematic Reviews

The main characteristics of the 13 included systematic reviews are summarized in Table 1. Each systematic review included the original trials, whose numbers ranged from 3 to 37. The included systematic reviews were published from 2010 onwards.

#### 3.1.3. Methodological Quality of Included Reviews

From the total of 13 systematic reviews, only two were rated as having “high” confidence. Two were rated as “low,” and the rest were rated as “critically low” confidence (Supplementary file II, AMSTAR2). In particular, no systematic reviews explained their selection of the study designs for inclusion, and the sources of funding for the included studies. Few studies (3/13) included PICO components and provided satisfactory explanations for, and discussion of, any heterogeneity observed in the results. Almost half of the reviews (6/13) performed data extraction in duplicate, while all performed study selection in duplicate and seemed to use a comprehensive literature search strategy.

### 3.2. Effects of Interventions

A summary of the main outcomes of the current umbrella review can be found in Table 2.

#### 3.2.1. Cancer-Related Fatigue

The effects of exercise and physical activity on fatigue was assessed in five systematic reviews [5,16,17,19,23]. According to Chang et al. [16], acute lymphoblastic leukemia (ALL) survivors aged between 6 and 14 years old had a mean reduction of 11% in fatigue from baseline (*p*-value not reported) (with no significant differences between pre-training and post-training) after a 12-week community-based exercise training program (low heterogeneity; *p* > 0.05). Participants with cancer (lymphoma, leukemia, and a central nervous system (CNS) germ cell tumor) that were between 14 and 18 years old had a statistically significant improvement in general fatigue levels (effect size = −0.76; 95% confidence interval; CI, [−1.35, −0.17]; *p* = 0.01) from baseline to 8 weeks of training and at a 3-month follow-up (low heterogeneity, *p* > 0.05). As for sleep/rest (effect size = −0.35; 95% CI −0.92 to 0.22); *p* = 0.23) and cognitive (effect size = −0.35; 95% CI −0.92 to 0.23; *p* = 0.24) fatigue, there were no significant differences (low heterogeneity, *p* > 0.05).

Braam et al. [5] found no significant differences between the intervention and control groups in general (SMD −0.04; 95% CI −0.88 to 0.8; *p*-value not reported), sleep/rest or cognitive (SMD 0.07; 95% CI −0.77 to 0.91; *p* = 0.86) fatigue after an exercise training program in children and young adults during treatment for childhood ALL (limited data, heterogeneity not reported). Bhardwaj et al. [15] assessed fatigue among children with cancer during active treatment or during post-chemotherapy treatment after aerobic training programs and physiotherapy practice sessions. They found lower fatigue scores (*p*-value not reported) among intervention groups but with no statistically significant differences (heterogeneity not reported). According to Coombs et al. [17], fatigue was decreased (*p*-value not reported) in children and adolescents with ALL undergoing acute chemotherapy after a 3-week aerobic training program (heterogeneity not reported). Fatigue was assessed by the Piper Fatigue Scale (PFS). Furthermore, exercise interventions that lasted from 2 weeks to 12 months and had a positive effect on fatigue during maintenance chemotherapy. These interventions ranged from twice monthly to seven times weekly, and 15–120 min per session.

Supervised exercise interventions significantly reduced fatigue (SMD = −0.44; *p* < 0.001) in children and adolescents with cancer during or after treatment (no important heterogeneity; I^2^ = 38%; *p* = 0.18) [23].

#### 3.2.2. Muscle Strength

Eight systematic reviews assessed muscle strength after exercise and physical activity interventions [5,6,17,19,20,22,23,24].

Wolin et al. reported strong evidence for a benefit (*p*-value not reported) on muscle strength in adult and pediatric hematological cancer survivors, especially when the training programs were conducted in the hospital setting [24]. Moreover, according to Grimshaw et al., exercise interventions provided a positive impact (*p*-value not reported) on muscle strength during the intense treatment phase in children and adolescents with cancer (heterogeneity not reported) [6]. The study by Braam et al. [5] found that strength training programs had significant beneficial effects for the back and leg strength but not for the knee and ankle strength in children and young adults during treatment for childhood cancer (SMD 0.07; 95% CI −0.77 to 0.91; *p* = 0.86) (heterogeneity not reported). Additionally, the inspiratory training program did not improve inspiratory muscle strength (heterogeneity not reported). In the study by Santos et al. [22], the muscle strength of upper and lower limbs of hospitalized children and adolescents with cancer was assessed after exercise programs. These programs mainly combined aerobic and strength training. They found that not only was muscle strength improved, (*p*-value not reported) but was also maintained for 20 weeks after the end of the exercise intervention (heterogeneity not reported).

An exercise intervention was able to improve strength (*p*-value not reported) during maintenance chemotherapy, post-treatment survivorship, and multiple phases of treatment in children and adolescents with ALL (heterogeneity not reported) [17]. The exercise program during maintenance chemotherapy lasted from 2 weeks to 12 months and included two sessions per month to seven sessions per week. The duration of the sessions was from 15 to 120 min. The exercise program during post-treatment survivorship lasted from 2 weeks to 4 months and included two sessions per month to seven sessions per week. The duration of the sessions was from 15 to 120 min. Lastly, the exercise program during multiple phases of treatment lasted from 12 to 135 weeks and included 1–2 sessions per month to seven sessions per week. The duration of the sessions was from 45 to 60 min.

Martha et al. [19] assessed the peripheral muscle strength of children and adolescents submitted to transplantation of hematopoietic stem cells after mixed exercise programs with aerobic and strength training. These programs lasted from 6 to 8 weeks with three to seven sessions per week. The duration of the sessions ranged from 20 to 120 min and the exercise intensity ranged from mild to moderate. There were benefits found regarding the peripheral muscle strength of these individuals that unfortunately were not clearly demonstrated (*p*-value not reported). However, positive responses were observed in relation to the analyzed data. The limitations were the high heterogeneity between the studies, as well as the sample size, and the low methodological rigor.

Shi et al. [23] found that supervised exercise interventions in childhood cancer survivors provided a significant enhancement in muscle strength (SMD = 1.42; 95% CI = 0.10~2.74; *p* = 0.03) (considerable heterogeneity; I2 = 95%; *p* < 0.001).

Interestingly, distance-delivered physical activity interventions could also collectively improve muscle strength (*p*-value not reported) in childhood cancer survivors (heterogeneity not reported) [20].

#### 3.2.3. Aerobic Capacity

Physical activity interventions improved aerobic capacity (*p*-value not reported) during the intense treatment phase in children and adolescents with cancer (heterogeneity not reported) [6]. Specially designed exercise programs led to submaximal aerobic capacity enhancement (*p*-value not reported) and endurance improvement (*p*-value not reported) in pediatric brain tumor survivors (heterogeneity not reported) [18].

#### 3.2.4. Cancer-Related Pain

Grimshaw et al. [6] have evaluated the impact of physical activity on reported pain induced by cancer during intensive treatment in children and adolescents with cancer and reported improvements (*p*-value not reported) in that symptom (heterogeneity not reported).

#### 3.2.5. Body Composition

The evidence for body composition after exercise programs in pediatric hematological cancer survivors who did not receive hematopoietic stem cell transplantation is unfortunately weak. This evidence showed that a 16-week combined aerobic and resistance training intervention of thrice weekly activity for 90–120 min did not improve body composition (*p*-value not reported) in very young (4–7 years of age) pediatric ALL survivors (heterogeneity not reported) [24].

After data analysis for body mass index (BMI), Braam et al. [5] found a non-significant intervention effect in children and young adults with ALL. There was no substantial heterogeneity (I^2^ = 48%) between the studies with a standardized mean difference (SMD) of 0.59 on the Quetelet index (95% CI −0.23 to 1.41; *p* = 0.16).

Morales et al. [8] found that training programs with a combination of aerobic and resistance exercises that lasted between 12 weeks and 6 months with three to five sessions per week led to a decrease in central adiposity (*p*-value not reported) in childhood cancer survivors (waist circumference and waist-to-hip ratio) (heterogeneity not reported). As for body weight, or BMI, most findings tended to support that exercise programs did not manage an improvement (*p*-value not reported) (heterogeneity not reported).

Supervised exercise interventions that included aerobic, anaerobic, resistance, or combined training regimens managed no significant effect on body composition in childhood cancer survivors (heterogeneity not reported) [23]. Moreover, supervised exercise significantly increased BMI in the intervention group compared to control group [mean differences (MD) = 1.06, 95% CI = 0.13~1.99, *p* = 0.03)] (substantial heterogeneity, I^2^ = 82%, *p* = 0.004). Sensitivity analysis confirmed that the results of BMI are robust and reliable [23].

#### 3.2.6. Activity and Participation Levels

Even though participation in physical activity and exercise is essential for the development of every child, it is decreased in childhood cancer patients [25]. Coombs et al. [17] reported that activity levels had increased (*p*-value not reported) during maintenance chemotherapy and multiple-phase interventions, while participation levels had increased (*p*-value not reported) only during multiple-phase interventions in children and adolescents with ALL who participated in a physical activity program (heterogeneity not reported). According to Braam et al. [5] there were no statistically significant differences (*p*-value not reported) between the intervention group, which followed physical activity interventions, and the control group, related to the level of daily activity in children and young adults with ALL during chemotherapy (heterogeneity not reported).

Distance-delivered physical activity interventions for childhood cancer survivors did not significantly increase (*p*-value not reported) self-reported physical activity levels, according to Mizrahi et al. [20] (heterogeneity not reported). Morales et al. [21] reported an increase (*p*-value not reported) in activity levels in childhood cancer survivors after exercise training programs (heterogeneity not reported). Activity levels were assessed by means of a questionnaire and an accelerometer. Khaleqi-Sohi et al. [18] assessed physical activity levels in pediatric brain tumor survivors after exercise interventions and reported improvements (*p*-value not reported) (heterogeneity not reported).

Supervised exercise interventions in childhood cancer survivors significantly improved the levels of daily physical activity (SMD = 1.05, *p* < 0.001) (substantial heterogeneity, I2 = 66%, *p* = 0.03). Levels of daily physical activity were measured using the Chinese University of Hong Kong Physical Activity Rating for Children and Youth scales, the German Momo questionnaire, or the acceleration for objective measurement [23].

#### 3.2.7. Psychosocial Health Indices

Physical activity interventions helped to improve self-esteem (*p*-value not reported) and mental health (*p*-value not reported) during the intense treatment phase for children and adolescents with cancer (heterogeneity not reported) [6]. Moreover, distance-delivered physical activity interventions collectively improved negative mood, interpersonal problems, self-esteem, ineffectiveness, anhedonia, and fatigue in childhood cancer survivors (heterogeneity not reported) (*p*-values not reported) [20].

Pediatric brain tumor survivors’ psychosocial health benefited from training programs that consisted of aerobic exercise or a combination of aerobic and strengthening exercises, or yoga. There was a decrease in depression levels (*p*-value not reported) which showed a positive correlation with the increased thickness of the cortex due to exercise (heterogeneity not reported) [18]. Depression levels were assessed by the Children’s Depression Inventory-2 questionnaire.

Supervised exercise interventions in childhood cancer survivors had a significant positive effect on self-efficacy (*p*-value not reported), according to Shi et al. [23] (heterogeneity not reported). For the measurement of self-efficacy, the Physical Activity Self-Efficacy Scale (PA-SE) was used.

#### 3.2.8. Health-Related Quality of Life

Physical activity interventions proved to have positive effects on health-related quality of life (HRQOL) scores at day 14 post-hematopoietic stem cell transplant (SMD = 2.24; 95% CI 1.35–3.13; *p*-value not reported) and at discharge (SMD = 1.86; 95% CI 0.97–2.75; *p*-value not reported) in children and adolescence with cancer, during the intense treatment phase, according to the Oncology module KINDL scale [6]. However, considering the generic version of KINDL, there was a negative effect on HRQOL at day 14 post-hematopoietic stem cell transplant (SMD = −1.64; 95% CI −0.75 to −2.53; *p*-value not reported) [6].

Health-related quality of life (QOL) was assessed after the completion of exercise interventions in children and young adults receiving treatment for ALL, or shortly after diagnosis. There were non-significant differences found for this outcome (*p*-value for data set not reported) (heterogeneity not reported) [5].

Santos et al. [22] reported that most data about the effects of exercise on QOL in children and adolescents with cancer during hospitalization showed non-significant differences (*p*-value not reported) (heterogeneity not reported).

The benefits of exercise interventions regarding QOL in children and adolescents submitted to transplantation of hematopoietic stem cells were not clearly demonstrated because analysis was limited. Nonetheless, positive responses were observed (*p*-value not reported) in relation to the analyzed data (heterogeneity not reported) [19]. The above exercise interventions consisted of exercise training programs with both aerobic and strength training.

Pediatric brain tumor survivors benefited (*p*-value not reported) after a 4-week inpatient rehabilitation and a 12-week yoga practice (heterogeneity not reported). Unfortunately, the positive impact did not last for a year [18]. The impact of interventions on QOL was assessed by the KINDL health-related quality of life and Peds 4.0 general module.

Supervised exercise interventions in childhood cancer survivors had no significant effect on QOL (SMD = 0.21; *p* = 0.20) (moderate heterogeneity, I^2^ = 53%, *p* = 0.08). For the evaluation of QOL after an exercise intervention program, the PedsQL 3.0 and 4.0, the German language KINDL questionnaire, and the “Vécu et Santé Perçue de l’Adolescent et de l’enfant” questionnaire (VSP-A) was used [23].

#### 3.2.9. Cardiorespiratory Fitness

Wolin et al. [24] found strong beneficial evidence (*p*-value not reported) regarding the effects of exercise in pediatric hematological cancer survivors (heterogeneity not reported).

Braam et al. [5] evaluated cardiorespiratory fitness after exercise training interventions in children and young adults during and after treatment for childhood cancer. They defined cardiorespiratory fitness as peak oxygen uptake (VO_2_peak), maximal power output (Wmax), or endurance time. Cardiorespiratory fitness was assessed by four tests. The combined 9-min run-walk test results showed significant differences between the intervention and the control groups, in favor of the intervention group (SMD = 0.69; 95% CI = 0.02–1.35) (moderate heterogeneity; I^2^ = 44%). The timed up and down stairs test showed no significant differences in cardiorespiratory fitness (SMD = −0.54; 95% CI = −1.77–0.70) (considerable heterogeneity; I^2^ = 84%), and both the 20-min shuttle run test and the timed up and go test showed positive results for cardiorespiratory fitness in favor of the intervention group.

The systematic review by Morales et al. [8] assessed the effects of aerobic training programs or programs that combine aerobic and resistance training on cardiorespiratory fitness in children and adolescents during treatment for cancer or in childhood cancer survivors. The meta-analysis showed a non-significant trend towards an improvement in VO_2_peak (MD = 1.97 mL∙kg^−1^∙min^−1^, 95% CI = 0.12–4.06, *p* = 0.065) after the end of the exercise programs (no signs of heterogeneity; Q = 4.633; I^2^ = 0%) (no signs of publication bias, *p* = 0.602).

In the systematic review by Shi et al. [23], half the data on the effect of supervised exercise interventions on cardiorespiratory fitness in childhood cancer survivors showed improvements (*p*-value not reported) (heterogeneity not reported). Cardiorespiratory fitness was evaluated by VO_2_peak, ventilatory threshold, and the 6-min walk test.

#### 3.2.10. Cardiovascular Fitness

Cardiovascular fitness outcomes were reported in two systematic reviews. Mizrahi et al. [20] reported that cardiovascular fitness was improved (*p*-value not reported) after distance-delivered physical activity interventions in childhood cancer survivors (heterogeneity not reported). Cardiovascular fitness was measured by gold standard cardiopulmonary exercise testing with gas analysis to determine maximal oxygen consumption (VO_2_max), the Progressive Aerobic Cardiovascular Endurance Run (PACER), and the submaximal 6-min walk test.

In the systematic review by Khaleqi-Sohi et al. [18] the effects of physical activity and exercise therapy on cardiovascular fitness of pediatric brain tumor survivors were assessed by a 6-min walk test. The results of this test showed that the distance the individuals covered in 6 min increased (*p*-value not reported) after a 12-week training (heterogeneity not reported).

#### 3.2.11. Cardiovascular Function and Structure

According to Morales et al. [21], exercise interventions provided benefits to the endothelial function of childhood cancer survivors (heterogeneity not reported). Furthermore, exercise interventions appear to exert a cardioprotective effect in childhood cancer survivors by improving or attenuating the decline of physical cardiovascular function [8]. More specifically, exercise helps to preserve the left ventricular ejection fraction from decline (*n* = 44, MD = 0.29%, 95% CI = −1.41–1.99, *p* = 0.738) with no signs of heterogeneity (Q = 1.811, I^2^ = 0%) and no signs of publication bias (*p* = 0.296).

#### 3.2.12. Flexibility/Range of Motion

Two systematic reviews assessed the effectiveness of exercise interventions on ankle dorsiflexion [5,24]. Wolin et al. [24] reported that there was weak evidence regarding ankle dorsiflexion in pediatric hematological cancer survivors. Braam et al. [5] found that there were no statistically significant differences between the intervention and control groups with the active ankle dorsiflexion test in children and young adults with ALL during chemotherapy who participated in exercise interventions (heterogeneity not reported). However, a significant positive effect was found for passive ankle dorsiflexion in favor of the intervention group (SMD = 0.69; 95% CI = 0.12–1.25) (heterogeneity not reported). In addition, limited data showed no statistically significant difference between the intervention and control groups regarding body flexibility (heterogeneity not reported). Body flexibility was assessed using the sit-and-reach distance test [5].

Although the data were limited, they showed that distance-delivered physical activity interventions can improve flexibility (*p*-value not reported) in childhood cancer survivors (heterogeneity not reported) [20].

Exercise and motor interventions managed to improve the range of motion (*p*-value not reported) in children with ALL undergoing maintenance chemotherapy, and in children with ALL during post-treatment survivorship (heterogeneity not reported) [17].

Khaleqi-Sohi et al. [18] reported that a 12-week yoga intervention helped increase hamstring flexibility (*p*-value not reported) in pediatric brain tumors out-patients (heterogeneity not reported).

Limited data showed that flexibility and balance were effectively improved (*p*-value not reported) after supervised exercise interventions in childhood cancer survivors (heterogeneity not reported). These two outcomes were measured by the sit-and-reach test and flamingo balance test [23].

#### 3.2.13. Coordination

Coombs et al. [17] reported that coordination after exercise and motor interventions in children and adolescents with ALL was improved (*p*-value not reported) during multiple-phases of medical interventions (heterogeneity not reported). The interventions lasted from 12 to 135 weeks and the frequency of training units varied from 1–2 times per month to seven times per week.

After a 12-week exercise intervention, the bilateral coordination increased (*p*-value not reported) while balance remained unchanged (*p*-value not reported) in pediatric brain tumor survivors (heterogeneity not reported). Improved performance was maintained even 12 weeks after the training ended [18]. The Bruininks-Oseretsky Test-2 (BOT-2) was used to measure the motor proficiency level of the participants with eight subtests, among which were the tests for bilateral coordination and upper-limb coordination.

#### 3.2.14. Physical Fitness

The effect of exercise on the physical fitness of children and adolescents with cancer during hospitalization was positive. Physical fitness was not only increased (*p*-value not reported) after the exercise interventions but was also maintained for 20 weeks after the end of the training program (heterogeneity not reported) [22]. This outcome was measured using cardiopulmonary exercise testing.

Khaleqi-Sohi et al. [18] reported that a 12-week yoga program improved physical fitness (*p*-value not reported) in pediatric brain tumor survivors. Physical fitness was evaluated by the timed up and go test (TUG), and it was found that the participants performed the test significantly faster after the end of the training program than before the start of it (heterogeneity not reported). Intrahospital, the training program was performed under the supervision of a physiotherapist or a kinesiologist, and home-based, under the supervision of the participant’s parents.

#### 3.2.15. Physical Function

Unfortunately, there was weak evidence considering the effects of exercise on physical function in pediatric hematological cancer survivors [24]. As for children and adolescents undergoing treatment for intense phase cancer, Grimshaw et al. [6] reported that large effect sizes were calculated for motor performance (*p*-value not reported) within the domain of physical function after their participation in physical activity interventions (heterogeneity not reported).

Distance-delivered physical activity interventions provided a positive effect on physical function (*p* = 0.008) for childhood cancer survivors, according to Mizrahi et al. [20] (heterogeneity not reported). Physical function included cardiovascular fitness, muscular strength, functional capacity, and flexibility.

Limited data support that there was no beneficial training effect of the exercise intervention on functional performance (*p*-value not reported) in pediatric patients with cancer (solid tumors) after participating in supervised exercise interventions (heterogeneity not reported) [23]. Physical function was analyzed using the TUG and timed up and down stairs (TUDS) tests.

#### 3.2.16. Functional Capacity

Physical activity interventions helped to improve the functional capacity (*p*-value not reported) of children and adolescents with cancer during hospitalization [22]. This improvement was maintained for 20 weeks after the end of the interventions. Functional capacity was assessed through the 6-min walk test and showed no difference between the intervention and control groups, according to the results of the meta-analysis. High heterogeneity presented in this test’s meta-analysis due to the great variability of the maximum distance traveled. Furthermore, functional capacity evaluated through the TUDS test showed significant improvements in favor of the intervention group.

According to Coombs et al. [17], functional capacity was improved (*p*-value not reported) in children and adolescents on post-treatment survivorship for ALL after participating in exercise and motor interventions (heterogeneity not reported).

Martha et al. [19] reported that physical activity might be favorable for the functional capacity of children and adolescents treated with hematopoietic stem cell transplantation. Exercise improved the functional capacity assessed by the TUDS test (MD = −1.23 [95% CI = 2.27 to −0.20, I^2^ = 0%]), but there was no significant effect for the 6-min walk test (MD = 44.63 [95% CI = −20.86–110.13, I^2^ = 83%]).

#### 3.2.17. Physical Capacity

Exercise improved or at least attenuated the decline (*p*-value not reported) of physical capacity (i.e., increased performance on the 6-min walk test, and showed a trend towards an increase in VO_2_peak) in childhood cancer survivors (heterogeneity not reported). Therefore, exercise also exerted a cardioprotective effect in this population [8].

#### 3.2.18. Biochemical Indicators

Biochemical indicators, such as hemoglobin and glycated hemoglobin (HbA1c) levels, was not improved (*p*-value not reported) after distance-delivered physical activity interventions in survivors of childhood cancer (heterogeneity not reported) [20].

#### 3.2.19. Bone Mineral Density

Two systematic reviews evaluated how exercise affected the amount of bone mineral in bone tissue in ALL pediatric patients. For children and young adults during and after treatment for childhood cancer, analysis showed a significant SMD of 1.07 for total body bone mineral density (BMD) (95% CI 0.48 to 1.66; *p* < 0.001) after an intervention of 24 months [5]. These findings revealed a large and significant positive intervention effect on total body BMD for the intervention group compared to the control group. For the measurement of BMD, a dual-energy X-ray absorptiometry (DXA) scan was used to determine its changes (lumbar spine and total body) in children with childhood ALL. Coombs et al. [17] reported an improvement in BMD (*p*-value not reported) in children and adolescents with ALL during maintenance chemotherapy (heterogeneity not reported). Furthermore, limited findings showed that children with different types of cancer had an increase in their BMD content (*p*-value not reported) and femoral neck bone mineral density after participating in exercise programs (heterogeneity not reported) [21].

#### 3.2.20. Brain Volume and Structure

Preliminary evidence was found regarding the benefits on brain volume and structure (*p*-value not reported) of childhood brain tumor survivors after their participation in exercise interventions. More specifically, these benefits detected white matter fractional anisotropy and hippocampal volume, cortical thickness, and white matter volume (heterogeneity not reported) [21].

Khaleqi-Sohi et al. [18] assessed the effects of physical activity and exercise interventions in childhood brain tumor survivors. They reported that the improvements in motor proficiency and physical fitness after exercise therapy were consistent with magnetic resonance imaging results, as an increase in the right somatosensory cortical thickness (*p*-value not reported) and in fractional anisotropy in the corpus callosum (*p*-value not reported), as well as on the right corticospinal pathway and on the cingulum (heterogeneity not reported). For the measurement, the examination of brain neural communication using magnetoencephalography (MEG) was utilized.

#### 3.2.21. Cognitive Function

Physical activity and exercise interventions improved the cognitive function of pediatric tumor survivors [18]. More specifically, the exercise group showed improvements in reaction time (*p*-value not reported) after 12 weeks of training that also continued for 12 weeks after training had ended (heterogeneity not reported). The Cambridge Neuropsychological Test Automated Battery was utilized to study the patients’ cognitive function.

#### 3.2.22. Energy Consumption

In the systematic review by Khaleqi-Sohi et al. [18], limited data showed that active video gaming made no noticeable changes on the energy consumption (*p*-value not reported) of pediatric brain tumor survivors (heterogeneity not reported).

#### 3.2.23. General Health Domain

Grimshaw et al. [6] evaluated the effects of physical activity interventions on the general health domain of children and adolescents undergoing intensive treatment for cancer. They reported that parents’ responses to the general health domain of the Child Health Questionnaire showed significant improvements (*p*-value not reported) (heterogeneity not reported). On the contrary, the children’s data for the same domain showed a non-significant finding in favor of physical activity (heterogeneity not reported).

#### 3.2.24. Adverse Effects

Eleven systematic reviews assessed the safety of exercise interventions in childhood cancer [5,6,8,16,17,19,20,21,22,23,24]. Wolin et al. [24] reported that exercise in pediatric hematological cancer survivors was safe. Moreover, training during the neutropenic phase after HSCT did not increase the risk of adverse effects. Chang et al. [16] found that non-pharmacological interventions aimed at improving fatigue in children and adolescents with cancer were feasible and safe. According to Grimshaw et al. [6], physical activity interventions during the intense treatment phase in children and adolescents with cancer were proven to be feasible, acceptable, and safe. Limited data show that children and young adults with ALL experienced no negative effects from physical exercise interventions [5]. No adverse effects were noted in childhood cancer survivors who took part in distance-delivered physical activity interventions (limited data) [20]. Morales et al. [21] mentioned that most data showed that exercise training had no adverse effects in childhood cancer survivors, yet in some cases it caused problems regarding tolerance or safety. Physical exercise during hospitalization in children and adolescents with cancer did not cause any adverse effects according to Santos et al. [22]. Few specific adverse effects were reported after children and adolescents with ALL participated in exercise and motor interventions, but none of them were related to harm or injury [17]. In addition, none of the above adverse effects were associated with motor interventions. There were no reported problems or health risks related to physical exercise in children and adolescents submitted to the transplantation of hematopoietic stem cells, according to Martha et al. [19]. Morales et al. [8] noted some adverse effects during exercise interventions in children with cancer. More specifically, an incident of patellar dislocation, a fall during an exercise session, and the feeling of headache, muscle soreness, fatigue, and hyperventilation. Finally, Shi et al. [23] found that most childhood cancer survivors who took part in supervised exercise interventions suffered no adverse effects. There were only a few minor negative incidents, which were falls and muscle soreness.

## 4. Discussion

### 4.1. Summary of Main Results and Overall Completeness and Applicability of Evidence

This umbrella review provides a comprehensive assessment of the effects of exercise and physical activity levels in childhood cancer patients and survivors. However, high-quality systematic reviews were limited. An extensive range of exercise approaches was applied in the included systematic reviews. The implemented exercise interventions were resistance training, aerobic training, or a combination of them, flexibility, functional, balance, motor skill training, physiotherapy practice sessions, yoga interventions, and physical activity games.

There were a great variety of health-related outcomes measured in the systematic reviews included in the present umbrella review. Many cancer patients suffer from severe fatigue during active treatment and in the survivorship phase [26], which also affects the quality of their everyday life [27]. The effects of exercise and physical activity on fatigue was assessed in five systematic reviews. Most reported that a wide range of exercise training programs had a beneficial effect on the fatigue levels of children with ALL or other types of cancer and on different phases of treatment [16,17]. However, two systematic reviews did not find any statistically significant differences for childhood patients with ALL undergoing acute chemotherapy [17] and for childhood cancer survivors [16].

A variety of strength exercise programs provided positive effects on children and adolescents who survived ALL or other types of cancer [20,23,24], and on children and adolescents during multiple phases of cancer treatment [5,6,17,19,22], while improvements in the aerobic capacity of pediatric cancer patients and survivors were reported after the completion of exercise and physical activity interventions [6,18]. Although pain is a common symptom in pediatric cancer [28], only one systematic review assessed the impact of physical activity on pain, reporting positive results [6].The effects of exercise intervention programs on body composition of childhood cancer patients and survivors were assessed in five systematic reviews. The BMI of children and young adults with ALL was not significantly improved [5]. As for the BMI of childhood cancer survivors, Shi et al. [23] reported significant improvements, while Morales et al. [21] did not. The same population showed a decrease in their central adiposity [21]. Childhood cancer survivors managed no significant effect on their body composition [23], but children and adolescents undergoing intense treatment had positive short- and long-term body composition outcomes [6].

Six systematic reviews evaluated how exercise and physical activity interventions affected the activity and participation levels of childhood cancer patients and survivors. Activity levels and participation were increased in ALL during maintenance, multiple-phase interventions and cancer survivors [17,18,21,23], whereas one study reported no effect [20]. Children and adolescents who have survived cancer are at a higher risk of developing emotional problems compared to their heathy peers [29]. All studies examining how exercise and physical activity affected the psychosocial health of children reported improvements in self-esteem and mental health, negative mood, interpersonal problems, ineffectiveness, self-efficacy, anhedonia, and fatigue [6,18,20,23].

The QOL that is affected by treatment-related symptoms and the family environment [30] is improved through physical activity programs in children and adolescents with cancer and in survivors of childhood cancer [5,6,18,19,22,23], although one study reported positive effects only after the evaluation with the Oncology module of the KINDL scale, when the generic version of the KINDL showed a negative effect on QOL [6].

Cardiovascular fitness, function, and structure, as well as endothelial function are improved [18,20,21,22], or their decline is attenuated by exerting a cardioprotective effect [8] as a result of exercise programs. Physical activity interventions had positive effects on flexibility [5,17,18,20,23], balance [23], and coordination [17,18] in children with different types of cancer and in childhood cancer survivors. Beneficial effects were also reported for the physical function/functional capacity in various types of children and adolescent cancers as a result of physical activity interventions [6,17,19,20,22], whereas limited data on solid tumor patients showed no benefit [23]. Bone mineral content and density were improved by exercise programs [5,17,21], whereas hemoglobin and HbA1c levels of pediatric cancer survivors were not affected [20].Two systematic reviews noted benefits on brain volume and structure in childhood brain tumor survivors after their participation in exercise interventions. Morales et al. [21] conducted a systematic review of these benefits on white matter fractional anisotropy, hippocampal volume, cortical thickness, and white matter volume. Khaleqi-Sohi et al. [18] reported an increase in the right somatosensory cortical thickness and fractional anisotropy (FA) in the corpus callosum, as well as in the right corticospinal pathway and on the cingulum. These results were consistent with the improvements in motor proficiency and physical fitness. Furthermore, in a systematic review, Khaleqi-Sohi [18] reported that the cognitive function of pediatric tumor survivors was improved after their participation on active video gaming.

Grimshaw et al. [6] evaluated the effects of physical activity interventions on the general health domain of children and adolescents undergoing intensive treatment for cancer. They reported that parents’ responses to the general health domain showed significant improvements, while the children’s data for the same domain showed a non-significant finding in favor of physical activity.

Eleven systematic reviews assessed the safety of exercise interventions in childhood cancer. Most reported that exercise and physical activity intervention during childhood cancer or at the survivor stage is feasible and safe [5,6,16,19,20,22,24]. The remaining four systematic reviews expressed some concerns regarding tolerance or safety [21], falls, and muscle soreness [8]. In addition, an incident of patellar dislocation and headache, fatigue, hyperventilation [8], and a few others that were not related to harm or injury were reported [17].

The role biomarkers in therapy management is very interesting. Biomarkers seem to be important for the rehabilitation approach in cancer patients and childhood cancer survivors [31,32]. Inflammation and p16 INK4a expression are associated with lower exercise capacity in childhood cancer survivors [31]. P16 INK4a is a biomarker of cellular senescence whose loss allows precancerous lesions to bypass senescence [33]. Moreover, physical activity induced significant biochemical perturbations in key molecules in patients with breast cancer [32]. However, it is important to tailor the rehabilitative strategies to the patient’s needs [32].

### 4.2. Quality of the Evidence

Some limitations in this umbrella review were the lack of heterogeneity data, as well as the overlap of original studies across systematic reviews. The assessment of overlapping showed that one or more of the same original studies were analyzed in 44.3% of all systematic reviews (overlapping of original studies, Supplement III). In addition, with regard to the methodological quality, the AMSTAR2 scores show that the majority of the systematic reviews were of critically low quality. Moreover, some systematic reviews lacked substantial data regarding the exercise intensity or duration that were important for the understanding of the exercise interventions’ outcomes.

### 4.3. Study Limitations

Another limitation was the lack of presentation of the direct correlations between the outcomes and a specific type of physical exercise (i.e., aerobic, resistance, or a combination approach), with related training characteristics such as intensity, duration, frequency, and intervention time, since the systematic reviews included did not separate this information for every outcome presented.

### 4.4. Potential Biases in the Review Process

We conducted a comprehensive algorithmic search on the Cochrane Library, PubMed, and Embase databases, as well as created alerts to track upcoming systematic reviews that met our eligibility criteria. Furthermore, we reviewed the references list of the eligible systematic reviews. Although it is possible that we missed a couple of studies through the hand-searching of the reference lists, due to the great overlap between the results of the different databases, it was unlikely that we would fail to find all the studies.

## 5. Conclusions

### 5.1. Implications for Practice

Exercise and physical activity interventions were found to have a positive effect on many health outcomes in children with cancer and in childhood cancer survivors (Table 1). The beneficial impact was most evident on fatigue, muscle strength, aerobic capacity, activity and participation levels, psychosocial health, cardiovascular fitness, cardiovascular function and structure, flexibility, physical fitness, functional capacity, coordination, bone mineral density, and brain volume and structure. Furthermore, these interventions proved to be feasible and safe, with a limited number of systematic reviews reporting some adverse effects. Specifically, the reported adverse effects were headache, fatigue, hyperventilation, falls, muscle soreness, and an incident of patellar dislocation. The above effects are related to tolerance or safety and may cause discomfort or even musculoskeletal injuries that will need physical recovery. Close supervision during the exercise intervention is needed to avoid these adverse effects.

### 5.2. Implications for Research

We suggest considering the outcomes of the current umbrella review in everyday clinical practice; however, these should be treated with caution when reported by systematic reviews of critically low quality.

Future research is needed to focus on high-quality trials with long-term follow-up and the optimal type of exercise, in addition to the duration of the exercise intervention. Equally important is the determination of the optimum duration and intensity of exercise sessions. The above characteristics are necessary to be determined in order for exercise and physical activity guidelines for this population to be formed. National or international multicenter studies should be strongly encouraged for this purpose. To achieve the above, doctors and families should become familiar with the beneficial effects of exercise/physical activity programs and their safe nature at all therapy phases.

## Figures and Tables

**Table 1:** Study Characteristics and outcomes.

Study	Assessment of Bias	Sample Demographics	Type of Cancer	Cancer Treatment-Stage of Cancer	Intervention Characteristics	Main Results
[24]	Critically low	− 4–18 y (and adults) − *n* = 209	Any type of hematological cancer	− Pediatric survivors not receiving hematopoietic stem cell transplantation − Pediatric survivors undergoing HSCT	Type: Aerobic or a combination of aerobic and resistance exercise Frequency: NR Intervention duration: Not receiving HSCT: 12–20 weeks; Receiving HSCT: 8 weeks Exercise duration: ranged from 30–120 min Intensity: NR Supervised: home-based, intrahospital	− Strong evidence for a benefit on muscle strength (particularly if training was conducted in the hospital setting) − Body composition: weak evidence − ↑ Cardiorespiratory fitness − Ankle dorsiflexion: weak evidence − Physical functioning: weak evidence − No adverse effects
[16]	Low	− 6–18 y − *n* = 155	ALL (the most common), solid tumors, AML, and lymphoma	− Maintenance stage of chemotherapy − Just received their first round of chemotherapy − Had completed two courses of treatment (4–8 weeks each) − Survivor stage	Type: home-based aerobic exercise using a video compact disc; use of a bicycle-style exerciser; aerobics and various types of physical activities and strength-building exercises Frequency: ranged from 2 to 3 days/week to twice/day Intervention duration: ranged from 2 days–16 weeks Exercise duration: ranged from 10–45 min Intensity: heart rate of >90% of the maximum heart rate (HR max), or the increase in the percentage of heart rate reserve (% HRR) of 40–60% Supervised: intrahospital, home-based, community	− ↓ Fatigue (11% mean reduction) after 12 weeks of training in 6–14 years for ALL survivors − ↓ General fatigue levels after 8 weeks of training and at a 3-month follow-up in 14–18-year-old patients with cancer − No adverse effects
[6]	Low	− 0–18 y − *n* = 278	Mixed cancer diagnoses, ALL, osteosarcoma or Ewing’s sarcoma of the lower limb, hematological cancer, solid tumor, hematological disorders	− Intense phase − Intense cancer treatment included hematopoietic stem cell transplantation and all treatment phases except the ‘maintenance phase’ of leukemia therapy	Type: aerobic, strengthening and stretching exercises, games, and yoga Frequency: ranged from 1 day/week to twice/day Intervention duration: ranged from 3 weeks–3 months Exercise duration: ranged from 15–60 min Intensity: NR Supervised: intrahospital supervised, home-based	− ↑ Muscle strength − ↑ Aerobic capacity − ↓ Pain − ↑ Role/social–physical, self-esteem, and mental health − HRQOL: ↑ when assessed by the Oncology Module KINDL scale, but ↓ when assessed by the generic version of the KINDL − ↑ Physical function − General health domain: parent’s responses-significant findings, child data-non-significant findings − No adverse effects
[5]	High	− <19 y at diagnosis − *n* = 171 (males: *n* = 98, females: *n* = 70) − Participants in the training program needed to be no more than 5 years from diagnosis	ALL	− During chemotherapy − During the maintenance treatment period − Shortly after diagnosis	Type: strength and inspiratory training Frequency: NR Intervention duration: ranged from 10 weeks–2 years Exercise duration: ranged from 15–60 min Intensity: NR Supervised: at least a home-based exercise program with guidance from a therapist of the treating hospital	− Fatigue, general fatigue: non-significant differences between the control and intervention groups (limited data) − Sleep/rest fatigue, cognitive fatigue: no intervention effect − ↑ back and leg strength combination score − BMI: no statistically significant differences − ↑ BMD for the intervention group compared to the control group − Activity levels: no statistically significant differences (limited data) − HRQOL: some positive effects in favor of the intervention group (assessed by the PedsQL Cancer Module) − ↑ Cardiorespiratory fitness (defined as: VO2 peak, Wmax, or endurance time) was significantly improved by the 9-min run-walk test, timed up and down stairs test, the timed up and go time test, and the 20-m shuttle run test, but not the timed up and down stairs test − Flexibility: ↑ Passive ankle dorsiflexion but no active ankle dorsiflexion and body flexibility − No adverse effects (limited data)
[15]	Critically low	− 0–18 y − *n* = 116 (55 of all participants in exercise interventions)	Cancer	− Active treatment − Post-chemotherapy and following treatment	Type: aerobics, pedaling a stationary bicycle, and physiotherapy practice sessions Frequency: NR Intervention duration: NR Exercise duration: NR Intensity: NR Supervised: intrahospital, home-based	− ↓ Fatigue scores among intervention groups (with no statistically significant differences)
[20]	Critically low	− < 18 y at diagnoses − *n* = 270 (54% females)	ALL, solid tumor survivors, brain tumor survivors, childhood cancer survivors of mixed diagnoses	− Maintenance chemotherapy > 20 years following intensive treatment −Within 5 years of treatment completion − More than 5 years following intensive treatment completion	Type: aerobic based only, combinations of aerobic, resistance, interval, functional, and flexibility training Frequency: ranged from daily to twice/week and from 60–420 min/week Intervention duration: ranged from 2 weeks–1 year Exercise duration: NR Intensity: between 40 and 70% of heart rate reserve and 66–90% of maximum heart rate, at a ‘moderate to vigorous’ intensity Supervised: ≥50% of the intervention was unsupervised, home-based, supervised in the treating clinic, or community-based	− Muscle strength: collectively improved − Activity levels: non-significantly increased self-reported physical activity levels − Psychosocial health indices: collectively improved negative mood, interpersonal problems, self-esteem, ineffectiveness, anhedonia, and fatigue − ↑ Cardiovascular fitness − ↑ Flexibility − Biochemical indicators: did not improve hemoglobin and HbA1c levels − No adverse effects (limited data)
[21]	Critically low	− 6–41 y (the age at diagnosis, the time since diagnosis, and the time since the end of treatment ranged from 0–15 y, from 1–22 y, and from 1–21 y) − *n* = 296 (intervention: *n* = 189, females: *n* = 96) (control: *n* = 107, females: *n* = 48)	Different types of childhood cancer (the most common being hematological malignancies (leukemia) and brain tumors in childhood cancer), and survivors who had finished anticancer therapy ≥ 1 year before the study	N/R	Type: aerobic or a combination of aerobic and resistance exercise Frequency: ranged from two to five sessions/week Intervention duration: from 8 weeks–6 months Exercise duration: N/R Intensity: 50–60% of one-repetition maximum for resistance exercise and between 40% of heart rate (HR) reserve and > 90% of maximum HR for aerobic exercise Supervised: NR	− Body composition: ↓ central adiposity (waist circumference and waist-to-hip ratio) − ↑ in total body bone mineral content and femoral neck bone mineral density − ↑ Activity levels − Cardiovascular function and structure: improved endothelial function − Brain volume and structure: benefits on white matter fractional anisotropy and hippocampal volume and on cortical thickness and white matter volume (preliminary evidence) − Some adverse effects or problems regarding tolerance or safety (limited data)
[22]	Critically low	− 4–18 y − *n* = 172	Solid or hematologic (most of them ALL) cancer with no previous organ transplantation, post radiotherapy/chemotherapy sessions, or medical contraindication for exercise (solid tumors, extracranial solid tumors, ALL, AA, ALCL, AML, MPD, hematological malignancy, rhabdomyosarcoma, neuroblastoma, undetermined)	N/R	Type: combination of strength and aerobic training, balance activities, stretching, and games Frequency: ranged from two to five sessions/week Intervention duration: from 3 to 22 weeks Exercise duration: ranged from 10–120 min Intensity: N/R Supervised: during hospitalization	− ↑ Muscle strength and maintained for 20 weeks after the end of the study − HRQOL: non-significant differences − ↑ Physical fitness and maintained for 20 weeks after the end of the study − Functional capacity: ↑ when measured by the TUDS test, but non-significant differences when measured by 6MWT − No adverse effects
[17]	Critically low	− 0–18 y − *n* = 508 (intervention: *n* = 282, control: *n* = 226)	ALL	Acute chemotherapy, maintenance chemotherapy, post-treatment survivorship, multiple phases	Type: Aerobic training, general strengthening and/or ankle dorsiflexor strengthening, gastrocsoleus stretching and/or general stretching, bone strengthening, balance training, and motor skill training Frequency: − Maintenance chemotherapy: 2 days/month–7 days/week − Post-treatment survivorship: 2 days/month–7 days/week − Across multiple phases of treatment: 1–2 days/month–7 days/week Intervention duration: − Acute chemotherapy: 3 weeks − Maintenance chemotherapy: 2 weeks–12 months − Post-treatment survivorship: 2 weeks–4 months − Across multiple phases of treatment: 12–135 weeks Exercise duration: − Maintenance chemotherapy: 15–120 min - Post-treatment survivorship: 15–120 min − Across multiple phases of treatment: 45–60 min Intensity: NR Supervised: NR	− Fatigue: ↓ after aerobic training intervention during acute chemotherapy and maintenance chemotherapy − Muscle strength: ↑ during maintenance chemotherapy, post-treatment survivorship and multiple-phase interventions − Activity levels: ↑ during maintenance chemotherapy and multiple-phase interventions − Participation: ↑ during multiple-phase interventions − Flexibility: ↑ range of motion during maintenance chemotherapy and post-treatment survivorship − Functional mobility: ↑ during post-treatment survivorship − Coordination: ↑ during multiple-phase interventions − Bone mineral density: ↑ during maintenance chemotherapy − Some specific adverse events (none of them reported harm, injury, or adverse effects associated with motor interventions)
[19]	Critically low	− 3–18 y − *n* = 91 (intervention: *n* = 45, control: *n* = 46)	Cancer, HSCT	HSCT	Type: mixed exercise program with aerobic and strength training Frequency: ranged from 3 to 7 days/week Intervention duration: from 6–8 weeks Exercise duration: ranged from 20–120 min Intensity: mild to moderate Supervised: predominantly or partially supervised	− Muscle strength: positive responses regarding peripheral muscle strength (not clearly demonstrated) − HRQOL: significant ↑ in children’s comfort and resilience − Functional capacity: significant ↑ in TUDS test but no difference in 6MWT − No adverse effects
[9]	Critically low	− 5–38 y (the age at diagnosis ranged from 0–15 y, the time since diagnosis from 1–22 y, and the time since the end of treatment of those with CCS who had already finished treatment from 0–21 y) − *n* = 697 (CCS: *n* = 669, healthy: *n* = 28)	Childhood cancer survivors, different types of childhood cancer (the most common being ALL)	During or after treatment	Type: aerobic or a combination of aerobic and resistance exercise Frequency: ranged from 1 to 6 days/week Intervention duration: from 3 weeks–2.5 years Exercise duration: NR Intensity: ranged from 50–60% of 1 repetition maximum for resistance exercise and between 50 and >90% heart	− Cardiorespiratory fitness: non-significant trend towards an improvement in peak oxygen uptake (VO2peak) − Cardiovascular function and structure: preserved left ventricular ejection fraction from decline − ↑ Physical capacity or attenuating the decline − Some adverse effects: a patella dislocation, a fall during an exercise session, headache, muscle soreness, fatigue, and hyperventilation during the exercise interventions
[18]	Critically low	− 1–23 (1–10 years from the conclusion Of the treatment, or 1–5 years from the conclusion of the treatment, or during treatment in two studies that include ages between 4–18) − *n* = 306	Pediatric brain tumors (hemispheric or posterior fossa brain tumors in most studies)	Patients or survivors: children who had undergone either cranial or craniospinal radiation therapy (a number of them had also undergone surgical operations with or without chemotherapy)	Type: aerobic exercise, combinations of aerobic and strengthening exercises, yoga Frequency: ranged from 2 to 5 days/week Intervention duration: from 12–24 weeks Exercise duration: NR Intensity: NR Supervised: − intrahospital: under the supervision of a physiotherapist or a kinesiologist − home-based: under the supervision of their parents	− Aerobic capacity: submaximal aerobic capacity enhancement and endurance improvement − ↑ Physical activity levels − Psychosocial health indices: ↓ depression level showed a positive correlation with the increased thickness of the cortex (assessed by the CDI-2 questionnaire) − ↑ HRQOL: after 4 weeks of inpatient rehabilitation and after 12 weeks of yoga intervention (but the result did not last for a year) (assessed by the KINDL health related quality of life and the Peds 4.0 General Module) − Cardiovascular fitness: the distance covered in 6 min increased after a 12-week training period (assessed by 6 min walk test) − ↑ Hamstring flexibility after a 12-week yoga intervention in pediatric cancer out-patients − Physical fitness: after a 12-week yoga program, the participants performed the TUG-3m test significantly faster than the pre-test − Coordination: after a 12-week exercise training, the bilateral coordination increased while balance remained unchanged (improved performance maintained even 12 weeks after the training had ended) (measured by the Bruininks-Oseretsky Test2) − Brain volume and structure: ▪ Increased right somatosensory cortical thickness ▪ Increased fractional anisotropy (FA) in the corpus callosum, in the right corticospinal pathway, and in the cingulum − Cognitive function: improvement of reaction time after 12 weeks of training that was maintained for 12 weeks after training had ended (measured by the Cambridge Neuropsychological Test Automated Battery) − Energy consumption: no noticeable changes with active video gaming [assessed by the Metabolic Equivalent Task (MET)]
[23]	High	− 4–18 y − *n* = 642 (intervention: *n* = 322, control: *n* = 320)	Childhood cancer survivors with mixed types of cancer	During or after treatment	Type: aerobic, anaerobic, resistance, or combined physical exercise training Frequency: mean 2.25 sessions/week Intervention duration: mean 16.6 weeks Exercise duration: mean 152.36 min Intensity: low, Medium, high Supervised: supervised by health professionals, medical staff, or coaches	− ↓ Fatigue − ↑ Muscle strength − ↑ BMI, but non-significant effects on body composition − ↑ Level of daily physical activity − ↑ self-efficacy − HRQOL: no significant effect − ↑ cardiopulmonary fitness − ↑ Flexibility and balance (limited data) − Physical function: no intervention effect in pediatric patients with solid tumors (limited data)

Risk of Bias Assessment was completed using the AMSTAR 2 checklist. HRQOL—health-related quality of life, ALL—acute lymphoblastic leukemia, AML—acute myeloid leukemia, AA—aplastic anemia, ALCL—anaplastic large cell lymphoma, MPD—myeloproliferative disorder, HSCT-hematopoietic stem cell transplantation, CCS—childhood cancer survivors, BMD—bone mineral density, TUG-3m test—timed up and go test. ↑ Increased, ↓ Decreased.

**Table 2:** Main outcomes summary.

Outcome (Number of Studies)	Effect	Study	Type of Cancer
**Fatigue (5)**	↓	[15,16,17,23]	ALL (the most common), solid tumors, AML, lymphoma, CCS with mixed types of cancer
	↔	[5]	ALL
**Physical function:**			
**Muscle strength (8)**	↑	[5,6,17,19,20,22,23,24]	Hematological cancer (ALL, AA, ALCL, AML, MPD), osteosarcoma or Ewing’s sarcoma of the lower limb, solid tumor, extracranial solid tumor, rhabdomyosarcoma, neuroblastoma, undetermined hematological disorders, hematopoietic stem cell transplantation, CCS with mixed types of cancer
**Flexibility (5)**	↑	[18,20,23]	ALL, brain tumors, CCS with mixed types of cancer
	↔	[5]	ALL
	Weak evidence	[24]	Hematological cancer
**Range of motion (1)**	↑	[16]	ALL (undergoing maintenance chemotherapy and during post-treatment survivorship)
**Coordination (2)**	↑	[17,18]	ALL, pediatric brain tumors
**Physical fitness (2)**	↑	[18,22]	Solid tumors, extracranial solid tumor, hematological cancer (ALL, AA, ALCL, AML, MPD), rhabdomyosarcoma, neuroblastoma, brain tumors
**Motor performance (4)**	↑	[6,20]	ALL, solid tumors, solid tumor survivors, brain tumor survivors, osteosarcoma or Ewing’s sarcoma of the lower limb, hematological cancer, hematological disorders, CCS with mixed types of cancer
	↔	[23]	CCS with mixed types of cancer
	Weak evidence	[24]	Hematological cancer
**Functional capacity (3)**	↑	[17,19,22]	ALL, solid tumors, extracranial solid tumor, hematological cancer (AA, ALCL, AML, MPD), rhabdomyosarcoma, neuroblastoma, hematopoietic stem cell transplantation
**Physical capacity (1)**	↑	[8]	Different types of childhood cancer, CCS with mixed types of cancer
**CRF:**			
**Aerobic capacity (2)**	↑	[6,18]	ALL, osteosarcoma or Ewing’s sarcoma of the lower limb, hematological cancer, solid tumor, hematological disorders, brain tumors
**Cardiorespiratory fitness (4)**	↑	[5,23,24]	Hematological cancer (ALL and others), CCS with mixed types of cancer
	↔	[8]	Different types of childhood cancer, CCS with mixed types of cancer
**Cardiovascular fitness (2)**	↑	[18,20]	ALL, brain tumors, CCS with mixed types of cancer (solid tumor survivors, brain tumor survivors, and others)
**Cardiovascular function and structure (2)**	↑	[8,22]	Different types of childhood cancer, hematological malignancies, brain tumors, CCS with mixed types of cancer
**Pain (1)**	↓	[6]	ALL, osteosarcoma or Ewing’s sarcoma of the lower limb, hematological cancer, solid tumor, hematological disorders
**Body Weight/composition:**			
**Body Composition (3)**	↔	[22,23]	Hematological cancer, brain tumors, CCS with mixed types of cancer
	Weak evidence	[24]	Hematological cancer
**BMI (2)**	↔	[5]	ALL
	↑	[23]	CCS with mixed types of cancer
**Activity/participation levels (6)**	↑	[16,18,22,23]	ALL, hematological cancer, brain tumors, CCS with mixed types of cancer
	↔	[5,20]	ALL, solid tumor survivors, brain tumor survivors, CCS with mixed types of cancer
**Energy consumption (1)**	↔	[18]	Brain tumors
**Psychosocial health indices (4)**	↑	[6,18,20,23]	ALL, osteosarcoma or Ewing’s sarcoma of the lower limb, hematological cancer, solid tumor, brain tumors, hematological disorders, CCS with mixed types of cancer (solid tumor survivors, brain tumor survivors and others)
**HRQL (6)**	↑	[5,6,18,19]	Hematological cancer (ALL and others), osteosarcoma or Ewing’s sarcoma of the lower limb, solid tumor, brain tumors, hematological disorders, hematopoietic stem cell transplantation
	↔	[22,23]	Solid tumors, extracranial solid tumor, hematological cancer (ALL, AA, ALCL, AML, MPD), rhabdomyosarcoma, neuroblastoma, CCS with mixed types of cancer
**Biochemical indicators (1)**	↔	[20]	ALL, CCS with mixed types of cancer (solid tumor survivors, brain tumor survivors)
**Bone mineral density (3)**	↑	[5,19,22]	ALL, hematological cancer, brain tumors
**Brain Volume/structure (2)**	↑	[18,22]	Hematological cancer, brain tumors
**General health domain (1)**	↔	[6]	ALL, osteosarcoma or Ewing’s sarcoma of the lower limb, hematological cancer, solid tumor, hematological disorders
**Cognitive function (1)**	↑	[18]	Brain tumors
**Adverse effects (11)**	No adverse effects	[5,6,16,19,20,22,23,24]	Hematological cancer (ALL, AML, AA, ALCL, MPD, lymphoma), solid tumors, osteosarcoma or Ewing’s sarcoma of the lower limb, hematological disorders, extracranial solid tumor, rhabdomyosarcoma, neuroblastoma, hematopoietic stem cell transplantation, CCS with mixed types of cancer (solid tumor survivors, brain tumor survivors)
	Some adverse effects	[8,17,22]	Hematological cancer (ALL and others), brain tumors, CCS with mixed types of cancer
	Not reported	[15,18]	Brain tumors, cancer in general

↑ Increased, ↓ Decreased, ↔ No change, ALL—acute lymphoblastic leukemia, AML—acute myelogenous leukemia, CCS—childhood cancer survivors, AA—aplastic anaemia, ALCL—anaplastic large-cell lymphoma, MPD—myeloproliferative disorders, CRF—cardiovascular/cardiorespiratory fitness, HbA1c—glycosylated hemoglobin, HRQoL—health-related quality of life.

## Data Availability

Reported data from the systematic reviews incorporated in the present umbrella review can be found in the original systematic reviews cited in the References section.

## Supplementary Materials

The following supporting information can be downloaded at: https://www.mdpi.com/article/10.3390/healthcare11060820/s1, [Supplement I: Algorithms of Pubmed, Cochrane and Embase and Overall Risk of Bias of Original Studies (Table S1); Supplement II: AMSTAR2 (excel file); Supplement III: Overlapping of Original Studies (excel file)].[34,35,36,37,38,39,40,41,42,43,44,45,46,47,48,49,50,51,52,53,54,55,56,57,58,59,60,61,62,63,64,65,66,67,68,69,70,71,72,73,74,75,76,77,78,79,80,81,82,83,84,85,86,87,88,89,90,91,92,93,94,95,96,97,98,99,100,101,102,103,104,105,106,107,108,109,110,111]
