# Supplementary material for: Effects of Exercise and Physical Activity Levels on Childhood Cancer: An Umbrella Review
Source: Healthcare (Basel). 2023 Mar 10;11(6):820. doi: 10.3390/healthcare11060820 (PMC10048410; doi:10.3390/healthcare11060820)
Supplement: Supplementary file 1 [file healthcare-11-00820-s001.zip › Supplement a⌐_Revised.pdf]

Supplementary file

Effects of exercise and physical activity levels on childhood cancer: an umbrella review

Christina Rapti,[1] Petros C. Dinas,[2] Costas Chryssanthopoulos,[1] Alexandra Mila[1] and Anastassios Philippou[1]

1Department of Physiology, Medical School, National and Kapodistrian University of Athens, Athens, 115 27, Greece; chrisrp@med.uoa.gr (C.R.); chryssan@phed.uoa.gr (C.C.); christogiannos13@gmail.com (A.M.)

[2]FAME Laboratory, Department of Physical Education and Sport Science, University of Thessaly, Trikala, 42100, Greece; petros.cdgmail.com

*Correspondence: tfilipou@med.uoa.gr

Table of Contents

Key word algorithm PubMed 3

Table S1. Overall risk of bias of original studies. 10

C2 General

Key word algorithm PubMed

100,(#13 AND #36 AND #98 AND #99),,,"(""Cancer""[Title/Abstract] OR ""pediatric cancer""[Title/Abstract] OR ""pediatric cancer survivors""[Title/Abstract] OR ""acute myeloid leukemia""[Title/Abstract] OR ""myeloid leukemia""[Title/Abstract] OR ""haematological cancer""[Title/Abstract] OR ""malignant haematological disorders""[Title/Abstract] OR ""solid tumor""[Title/Abstract] OR ""non central nervous system cancer""[Title/Abstract] OR ""central nervous system cancer""[Title/Abstract] OR ""Neoplasms""[Title/Abstract] OR ""cancer survivors""[Title/Abstract]) AND (""Exercise""[Title/Abstract] OR ""exercise training""[Title/Abstract] OR ""high intensity training""[Title/Abstract] OR ""hit training""[Title/Abstract] OR ""high intensity interval training""[Title/Abstract] OR ""HIIT""[Title/Abstract] OR ""aerobic exercise""[Title/Abstract] OR ""aerobic training""[Title/Abstract] OR ""endurance training""[Title/Abstract] OR ""strength training""[Title/Abstract] OR ""resistance training""[Title/Abstract] OR ""weight training""[Title/Abstract] OR ""continuous exercise""[Title/Abstract] OR ""intermittent exercise""[Title/Abstract] OR ""isometric exercise""[Title/Abstract] OR ""plyometric exercise""[Title/Abstract] OR ""low intensity exercise""[Title/Abstract] OR ""low intensity exercise training""[Title/Abstract] OR ""high intensity exercise""[Title/Abstract] OR ""high intensity exercise training""[Title/Abstract] OR ""moderate intensity exercise""[Title/Abstract] OR ""exercise based rehabilitation""[Title/Abstract]) AND (""VO2""[Title/Abstract] OR ""maximum oxygen consumption""[Title/Abstract] OR ""VO2max""[Title/Abstract] OR ""peak oxygen consumption""[Title/Abstract] OR ""VO2peak""[Title/Abstract] OR ""quality of life""[Title/Abstract] OR ""QOL""[Title/Abstract] OR ""health related quality of life""[Title/Abstract] OR ""HRQOL""[Title/Abstract] OR ""hospital admissions""[Title/Abstract] OR ""Hospitalisation""[Title/Abstract] OR ""Hospitalization""[Title/Abstract] OR ""Rehospitalisation""[Title/Abstract] OR ""Rehospitalization""[Title/Abstract] OR ""Fatigue""[Title/Abstract] OR ""muscle strength""[Title/Abstract] OR ""muscular strength""[Title/Abstract] OR ""Strength""[Title/Abstract] OR ""Feasibility""[Title/Abstract] OR ""aerobic capacity""[Title/Abstract] OR ""Pain""[Title/Abstract] OR ""Flexibility""[Title/Abstract] OR ""Balance""[Title/Abstract] OR ""gait impairments""[Title/Abstract] OR ""functional mobility""[Title/Abstract] OR ""body composition""[Title/Abstract] OR ""body mass index""[Title/Abstract] OR ""BMI""[Title/Abstract] OR ""body fat""[Title/Abstract] OR ""motor performance""[Title/Abstract] OR ""activity energy expenditure""[Title/Abstract] OR ""level of physical activity""[Title/Abstract] OR ""Adherence""[Title/Abstract] OR ""immune function""[Title/Abstract] OR ""immune system""[Title/Abstract] OR ""immune parameters""[Title/Abstract] OR ""inflammation markers""[Title/Abstract] OR ""inflammatory markers""[Title/Abstract] OR ""cardiorespiratory fitness""[Title/Abstract] OR ""physical fitness""[Title/Abstract] OR ""physical activity""[Title/Abstract] OR ""cardiovascular function""[Title/Abstract] OR ""cardiovascular structure""[Title/Abstract] OR 80[UID] OR ""bone mineral density""[Title/Abstract] OR ""Coordination""[Title/Abstract] OR ""risk of infection""[Title/Abstract] OR ""exercise capacity""[Title/Abstract] OR ""Endurance""[Title/Abstract] OR ""Sleep""[Title/Abstract] OR ""motor function""[Title/Abstract] OR ""ankle dorsiflexion""[Title/Abstract] OR ""general health domain""[Title/Abstract] OR ""psychosocial health""[Title/Abstract] OR ""functional capacity""[Title/Abstract] OR ""body structure""[Title/Abstract] OR ""body function""[Title/Abstract] OR ""Activity""[Title/Abstract] OR ""Participation""[Title/Abstract] OR ""Freedom""[Title/Abstract] OR ""adverse effects""[Title/Abstract]) AND ""systematic review""[Title/Abstract]",682,09:13:04

99,Systematic review[Title/Abstract],,,"""systematic review""[Title/Abstract]","250,484",09:12:00

98,(#37 OR #38 OR #39 OR #40 OR #41 OR #42 OR #43 OR #44 OR #45 OR #46 OR #47 OR #48 OR #49 OR #50 OR #51 OR #52 OR #53 OR #54 OR #55 OR #56 OR #57 OR #58 OR #59 OR #60 OR #61 OR #62 OR #63 OR #64 OR #65 OR #66 OR #67 OR #68 OR #69 OR #70 OR #71 OR #72 OR #73 OR #74 OR #75 OR #76 OR #77 OR #78 OR #79 OR # 80 OR #81 OR #82 OR #83 OR #84 OR #85 OR #86 OR #87 OR #88 OR #89 OR #90 OR #91 OR #92 OR #93 OR #94 OR #95 OR #96 OR #97),,,"""VO2""[Title/Abstract] OR ""maximum oxygen consumption""[Title/Abstract] OR ""VO2max""[Title/Abstract] OR ""peak oxygen consumption""[Title/Abstract] OR ""VO2peak""[Title/Abstract] OR ""quality of life""[Title/Abstract] OR ""QOL""[Title/Abstract] OR ""health related quality of life""[Title/Abstract] OR ""HRQOL""[Title/Abstract] OR ""hospital admissions""[Title/Abstract] OR ""Hospitalisation""[Title/Abstract] OR ""Hospitalization""[Title/Abstract] OR ""Rehospitalisation""[Title/Abstract] OR ""Rehospitalization""[Title/Abstract] OR ""Fatigue""[Title/Abstract] OR ""muscle strength""[Title/Abstract] OR ""muscular strength""[Title/Abstract] OR ""Strength""[Title/Abstract] OR ""Feasibility""[Title/Abstract] OR ""aerobic capacity""[Title/Abstract] OR ""Pain""[Title/Abstract] OR ""Flexibility""[Title/Abstract] OR ""Balance""[Title/Abstract] OR ""gait impairments""[Title/Abstract] OR ""functional mobility""[Title/Abstract] OR ""body composition""[Title/Abstract] OR ""body mass index""[Title/Abstract] OR ""BMI""[Title/Abstract] OR ""body fat""[Title/Abstract] OR ""motor performance""[Title/Abstract] OR ""activity energy expenditure""[Title/Abstract] OR ""level of physical activity""[Title/Abstract] OR ""Adherence""[Title/Abstract] OR ""immune function""[Title/Abstract] OR ""immune system""[Title/Abstract] OR ""immune parameters""[Title/Abstract] OR ""inflammation markers""[Title/Abstract] OR ""inflammatory markers""[Title/Abstract] OR ""cardiorespiratory fitness""[Title/Abstract] OR ""physical fitness""[Title/Abstract] OR ""physical activity""[Title/Abstract] OR ""cardiovascular function""[Title/Abstract] OR ""cardiovascular structure""[Title/Abstract] OR 80[UID] OR ""bone mineral density""[Title/Abstract] OR ""Coordination""[Title/Abstract] OR ""risk of infection""[Title/Abstract] OR ""exercise capacity""[Title/Abstract] OR ""Endurance""[Title/Abstract] OR ""Sleep""[Title/Abstract] OR ""motor function""[Title/Abstract] OR ""ankle dorsiflexion""[Title/Abstract] OR ""general health domain""[Title/Abstract] OR ""psychosocial health""[Title/Abstract] OR ""functional capacity""[Title/Abstract] OR ""body structure""[Title/Abstract] OR ""body function""[Title/Abstract] OR ""Activity""[Title/Abstract] OR ""Participation""[Title/Abstract] OR ""Freedom""[Title/Abstract] OR ""adverse effects""[Title/Abstract]","6,121,696",09:10:30

97,Adverse effects[Title/Abstract],,,"""adverse effects""[Title/Abstract]","160,800",08:55:07

96,Freedom[Title/Abstract],,,"""Freedom""[Title/Abstract]","55,957",08:54:47
95,Participation[Title/Abstract],,,"""Participation""[Title/Abstract]","178,991",08:54:27
94,Activity[Title/Abstract],,,"""Activity""[Title/Abstract]","3,015,066",08:54:02
93,Body function[Title/Abstract],,,"""body function""[Title/Abstract]","1,368",08:53:44
92,Body structure[Title/Abstract],,,"""body structure""[Title/Abstract]","1,007",08:53:24
91,Functional capacity[Title/Abstract],,,"""functional capacity""[Title/Abstract]","15,781",08:52:26
90,Psychosocial health[Title/Abstract],,,"""psychosocial health""[Title/Abstract]","2,185",08:52:09
89,General health domain[Title/Abstract],,,"""general health domain""[Title/Abstract]",83,08:51:47
88,Ankle dorsiflexion[Title/Abstract],,,"""ankle dorsiflexion""[Title/Abstract]","2,612",08:49:54
87,Motor function[Title/Abstract],,,"""motor function""[Title/Abstract]","27,786",08:49:38
86,Sleep[Title/Abstract],,,"""Sleep""[Title/Abstract]","204,237",08:49:21
85,Endurance[Title/Abstract],,,"""Endurance""[Title/Abstract]","36,415",08:49:05
84,Exercise capacity[Title/Abstract],,,"""exercise capacity""[Title/Abstract]","15,437",08:46:00
83,Risk of infection[Title/Abstract],,,"""risk of infection""[Title/Abstract]","17,438",08:45:00
82,Coordination[Title/Abstract],,,"""Coordination""[Title/Abstract]","134,119",08:44:39
81,Bone mineral density[Title/Abstract],,,"""bone mineral density""[Title/Abstract]","47,073",08:43:52
80,Range of motion[Title/Abstract],,,"""range of motion""[Title/Abstract]","42,307",08:43:36
79,Cardiovascular structure[Title/Abstract],,,"""cardiovascular structure""[Title/Abstract]",413,08:43:15
78,Cardiovascular function[Title/Abstract],,,"""cardiovascular function""[Title/Abstract]","7,529",08:42:58
77,Physical activity[Title/Abstract],,,"""physical activity""[Title/Abstract]","141,553",08:42:43
76,Physical fitness[Title/Abstract],,,"""physical fitness""[Title/Abstract]","12,949",08:42:18
75,Cardiorespiratory fitness[Title/Abstract],,,"""cardiorespiratory fitness""[Title/Abstract]","7,141",08:42:00
74,Inflammatory markers[Title/Abstract],,,"""inflammatory markers""[Title/Abstract]","27,238",08:41:42
73,Inflammation markers[Title/Abstract],,,"""inflammation markers""[Title/Abstract]","3,408",08:41:27
72,Immune parameters[Title/Abstract],,,"""immune parameters""[Title/Abstract]","3,848",08:41:10
71,Immune system[Title/Abstract],,,"""immune system""[Title/Abstract]","137,916",08:40:48
70,Immune function[Title/Abstract],,,"""immune function""[Title/Abstract]","24,983",08:40:27
69,Adherence[Title/Abstract],,,"""Adherence""[Title/Abstract]","151,202",08:40:10
68,Level of physical activity[Title/Abstract],,,"""level of physical activity""[Title/Abstract]","3,998",08:39:52
67,Activity energy expenditure[Title/Abstract],,,"""activity energy expenditure""[Title/Abstract]",767,08:39:31
66,Motor performance[Title/Abstract],,,"""motor performance""[Title/Abstract]","9,327",08:39:11
65,Body fat[Title/Abstract],,,"""body fat""[Title/Abstract]","36,978",08:38:42
64,BMI[Title/Abstract],,,"""BMI""[Title/Abstract]","185,211",08:38:22
63,Body mass index[Title/Abstract],,,"""body mass index""[Title/Abstract]","227,792",08:37:35
62,Body composition[Title/Abstract],,,"""body composition""[Title/Abstract]","44,817",08:36:57
61,Functional mobility[Title/Abstract],,,"""functional mobility""[Title/Abstract]","2,099",08:36:24
60,Gait impairments[Title/Abstract],,,"""gait impairments""[Title/Abstract]",478,08:35:35
59,Balance[Title/Abstract],,,"""Balance""[Title/Abstract]","267,181",08:34:24
58,Flexibility[Title/Abstract],,,"""Flexibility""[Title/Abstract]","92,738",08:34:01
57,Pain[Title/Abstract],,,"""Pain""[Title/Abstract]","745,484",08:33:34
56,Aerobic capacity[Title/Abstract],,,"""aerobic capacity""[Title/Abstract]","6,232",08:33:13
55,Feasibility[Title/Abstract],,,"""Feasibility""[Title/Abstract]","231,717",08:31:35
54,Strength[Title/Abstract],,,"""Strength""[Title/Abstract]","360,659",08:31:10
53,Muscular strength[Title/Abstract],,,"""muscular strength""[Title/Abstract]","4,236",08:30:32
52,Muscle strength[Title/Abstract],,,"""muscle strength""[Title/Abstract]","28,301",08:29:50
51,Fatigue[Title/Abstract],,,"""Fatigue""[Title/Abstract]","119,349",07:53:34
50,Rehospitalization[Title/Abstract],,,"""Rehospitalization""[Title/Abstract]","6,973",07:52:02
49,Rehospitalisation[Title/Abstract],,,"""Rehospitalisation""[Title/Abstract]",862,07:51:00
48,Hospitalization[Title/Abstract],,,"""Hospitalization""[Title/Abstract]","160,753",07:50:32
47,Hospitalisation[Title/Abstract],,,"""Hospitalisation""[Title/Abstract]","20,229",07:49:59
46,Hospital admissions[Title/Abstract],,,"""hospital admissions""[Title/Abstract]","21,081",07:49:19
45,HRQOL[Title/Abstract],,,"""HRQOL""[Title/Abstract]","22,607",07:48:50
44,Health related quality of life[Title/Abstract],,,"""health related quality of life""[Title/Abstract]","56,651",07:48:32
43,QOL[Title/Abstract],,,"""QOL""[Title/Abstract]","50,091",07:48:07
42,Quality of life[Title/Abstract],,,"""quality of life""[Title/Abstract]","348,301",07:47:48
41,VO2peak[Title/Abstract],,,"""VO2peak""[Title/Abstract]","4,688",07:47:20
40,Peak oxygen consumption[Title/Abstract],,,"""peak oxygen consumption""[Title/Abstract]","3,444",07:46:58

C2 General

39,VO2max[Title/Abstract],,,"""VO2max""[Title/Abstract]","12,012",07:46:35
38,Maximum oxygen consumption[Title/Abstract],,,"""maximum oxygen consumption""[Title/Abstract]","1,166",07:45:33
37,VO2[Title/Abstract],,,"""VO2""[Title/Abstract]","19,777",07:44:41
36,(#14 OR #15 OR #16 OR #17 OR #18 OR #19 OR #20 OR #21 OR #22 OR #23 OR #24 OR #25 OR #26 OR #27 OR #28 OR #29 OR #30 OR #31 OR #32 OR #33 OR #34 OR #35),,,"""Exercise""[Title/Abstract] OR ""exercise training""[Title/Abstract] OR ""high intensity training""[Title/Abstract] OR ""hit training""[Title/Abstract] OR ""high intensity interval training""[Title/Abstract] OR ""HIIT""[Title/Abstract] OR ""aerobic exercise""[Title/Abstract] OR ""aerobic training""[Title/Abstract] OR ""endurance training""[Title/Abstract] OR ""strength training""[Title/Abstract] OR ""resistance training""[Title/Abstract] OR ""weight training""[Title/Abstract] OR ""continuous exercise""[Title/Abstract] OR ""intermittent exercise""[Title/Abstract] OR ""isometric exercise""[Title/Abstract] OR ""plyometric exercise""[Title/Abstract] OR ""low intensity exercise""[Title/Abstract] OR ""low intensity exercise training""[Title/Abstract] OR ""high intensity exercise""[Title/Abstract] OR ""high intensity exercise training""[Title/Abstract] OR ""moderate intensity exercise""[Title/Abstract] OR ""exercise based rehabilitation""[Title/Abstract]","322,176",07:43:43
35,Exercise based rehabilitation[Title/Abstract],,,"""exercise based rehabilitation""[Title/Abstract]",232,07:37:34
34,Moderate intensity exercise[Title/Abstract],,,"""moderate intensity exercise""[Title/Abstract]","2,233",07:37:04
33,High intensity exercise training[Title/Abstract],,,"""high intensity exercise training""[Title/Abstract]",230,07:36:38
32,High intensity exercise[Title/Abstract],,,"""high intensity exercise""[Title/Abstract]","3,290",07:36:02
31,Low intensity exercise training[Title/Abstract],,,"""low intensity exercise training""[Title/Abstract]",84,07:35:38
30,Low intensity exercise[Title/Abstract],,,"""low intensity exercise""[Title/Abstract]","1,115",07:35:17
29,Plyometric exercise[Title/Abstract],,,"""plyometric exercise""[Title/Abstract]",181,07:28:16
28,Isometric exercise[Title/Abstract],,,"""isometric exercise""[Title/Abstract]","1,804",07:27:52
27,Intermittent exercise[Title/Abstract],,,"""intermittent exercise""[Title/Abstract]","1,234",07:27:02
26,Continuous exercise[Title/Abstract],,,"""continuous exercise""[Title/Abstract]",851,07:26:27
25,Weight training[Title/Abstract],,,"""weight training""[Title/Abstract]","1,151",07:26:04
24,Resistance training[Title/Abstract],,,"""resistance training""[Title/Abstract]","10,850",07:24:03
23,Strength training[Title/Abstract],,,"""strength training""[Title/Abstract]","6,486",07:23:43
22,Endurance training[Title/Abstract],,,"""endurance training""[Title/Abstract]","5,355",07:23:09
21,Aerobic training[Title/Abstract],,,"""aerobic training""[Title/Abstract]","3,083",07:22:51
20,Aerobic exercise[Title/Abstract],,,"""aerobic exercise""[Title/Abstract]","12,031",07:22:23
19,HIIT[Title/Abstract],,,"""HIIT""[Title/Abstract]","1,929",07:19:08
18,High intensity interval training[Title/Abstract],,,"""high intensity interval training""[Title/Abstract]","2,749",07:18:51
17,HIT training[Title/Abstract],,,"""hit training""[Title/Abstract]",22,07:18:26
16,High intensity training[Title/Abstract],,,"""high intensity training""[Title/Abstract]",935,07:18:07
15,Exercise training[Title/Abstract],,,"""exercise training""[Title/Abstract]","20,308",07:17:49
14,Exercise[Title/Abstract],,,"""Exercise""[Title/Abstract]","312,451",07:03:02
13,(#1 OR #2 OR #3 OR #4 OR #5 OR #6 OR #7 OR #8 OR #9 OR #10 OR #11 OR #12),,,"""Cancer""[Title/Abstract] OR ""pediatric cancer""[Title/Abstract] OR ""pediatric cancer survivors""[Title/Abstract] OR ""acute myeloid leukemia""[Title/Abstract] OR ""myeloid leukemia""[Title/Abstract] OR ""haematological cancer""[Title/Abstract] OR ""malignant haematological disorders""[Title/Abstract] OR ""solid tumor""[Title/Abstract] OR ""non central nervous system cancer""[Title/Abstract] OR ""central nervous system cancer""[Title/Abstract] OR ""Neoplasms""[Title/Abstract] OR ""cancer survivors""[Title/Abstract]","2,310,171",07:01:26
12,Cancer survivors[Title/Abstract],,,"""cancer survivors""[Title/Abstract]","21,364",06:56:22
11,Neoplasms[Title/Abstract],,,"""Neoplasms""[Title/Abstract]","241,358",06:55:59
10,Central nervous system cancer[Title/Abstract],,,"""central nervous system cancer""[Title/Abstract]",174,06:55:26
9,Non-central nervous system cancer[Title/Abstract],,,"""non central nervous system cancer""[Title/Abstract]",29,06:53:54
8,Solid tumor[Title/Abstract],,,"""solid tumor""[Title/Abstract]","13,994",06:53:17
7,Malignant haematological disorders[Title/Abstract],,,"""malignant haematological disorders""[Title/Abstract]",62,06:52:48
6,Haematological cancer[Title/Abstract],,,"""haematological cancer""[Title/Abstract]",393,06:41:08
5,Myeloid leukemia[Title/Abstract],,,"""myeloid leukemia""[Title/Abstract]","53,834",06:40:39
4,Acute myeloid leukemia[Title/Abstract],,,"""acute myeloid leukemia""[Title/Abstract]","36,761",06:39:54
3,Pediatric cancer survivors[Title/Abstract],,,"""pediatric cancer survivors""[Title/Abstract]",354,06:39:15
2,Pediatric cancer[Title/Abstract],,,"""pediatric cancer""[Title/Abstract]","4,475",06:38:36
1,Cancer[Title/Abstract],,,"""Cancer""[Title/Abstract]","2,085,814",06:38:15

Key word algorithm Cochrane

ID Search

C2 General

#1 Cancer
#2 Pediatric cancer
#3 Pediatric cancer survivors
#4 Acute myeloid leukemia
#5 Myeloid leukemia
#6 Haematological cancer
#7 Malignant haematological disorders
#8 Solid tumor
#9 Non-central nervous system cancer
#10 Central nervous system cancer
#11 Neoplasms
#12 Cancer survivors
#13 {OR #1-#12}
#14 Exercise
#15 Exercise training
#16 High intensity training
#17 HIT training
#18 High intensity interval training
#19 HIIT
#20 Aerobic exercise
#21 Aerobic training
#22 Endurance training
#23 Strength training
#24 Resistance training
#25 Weight training
#26 Continuous exercise
#27 Intermittent exercise
#28 Isometric exercise
#29 Plyometric exercise
#30 Low intensity exercise
#31 Low intensity exercise training
#32 High intensity exercise
#33 High intensity exercise training
#34 Moderate intensity exercise
#35 Exercise based rehabilitation
#36 {OR #14-#35}
#37 VO2
#38 Maximum oxygen consumption
#39 VO2max
#40 Peak oxygen consumption
#41 VO2peak
#42 Quality of life
#43 QOL
#44 Health related quality of life
#45 HRQOL
#46 Hospital admissions
#47 Hospitalisation
#48 Hospitalization
#49 Rehospitalisation
#50 Rehospitalization
#51 Fatigue
#52 Muscle strength
#53 Muscular strength
#54 Strength
#55 Feasibility
#56 Aerobic capacity
#57 Pain

C2 General

#58	Flexibility
#59	Balance
#60	Gait impairments
#61	Functional mobility
#62	Body composition
#63	Body mass index
#64	BMI
#65	Body fat
#66	Motor performance
#67	Activity energy expenditure
#68	Level of physical activity
#69	Adherence
#70	Immune function
#71	Immune system
#72	Immune parameters
#73	Inflammation markers
#74	Inflammatory markers
#75	Cardiorespiratory fitness
#76	Physical fitness
#77	Physical activity
#78	Cardiovascular function
#79	Cardiovascular structure
#80	Range of motion
#81	Bone mineral density
#82	Coordination
#83	Risk of infection
#84	Exercise capacity
#85	Endurance
#86	Sleep
#87	Motor function
#88	Ankle dorsiflexion
#89	General health domain
#90	Psychosocial health
#91	Functional capacity
#92	Body structure
#93	Body function
#94	Activity
#95	Participation
#96	Freedom
#97	Adverse effects
#98	{OR #37-#97}
#99	Systematic review
#100	{AND #13, #36, #98, #99}

Key word algorithm Embase

1	Cancer.ab,ti.	2828140
2	Pediatric cancer.ab,ti.	6962
3	Pediatric cancer survivors.ab,ti.	613
4	Acute myeloid leukemia.ab,ti.	63094
5	Myeloid leukemia.ab,ti.	87782
6	Haematological cancer.ab,ti.	625
7	Malignant haematological disorders.ab,ti.	82
8	Solid tumor.ab,ti.	22368

C2 General

9	Non-central nervous system cancer.ab,ti.	33
10	Central nervous system cancer.ab,ti.	223
11	Neoplasms.ab,ti.	124038
12	Cancer survivors.ab,ti.	32074
13	1 or 2 or 3 or 4 or 5 or 6 or 7 or 8 or 9 or 10 or 11 or 12	2999788
14	Exercise.ab,ti.	391536
15	Exercise training.ab,ti.	26693
16	High intensity training.ab,ti.	1045
17	HIT training.ab,ti.	31
18	High intensity interval training.ab,ti.	3172
19	HIIT.ab,ti.	2331
20	Aerobic exercise.ab,ti.	15926
21	Aerobic training.ab,ti.	4288
22	Endurance training.ab,ti.	6441
23	Strength training.ab,ti.	7435
24	Resistance training.ab,ti.	11719
25	Weight training.ab,ti.	1218
26	Continuous exercise.ab,ti.	1016
27	Intermittent exercise.ab,ti.	1252
28	Isometric exercise.ab,ti.	1977
29	Plyometric exercise.ab,ti.	138
30	Low intensity exercise.ab,ti.	1374
31	Low intensity exercise training.ab,ti.	105
32	High intensity exercise.ab,ti.	3866
33	High intensity exercise training.ab,ti.	323
34	Moderate intensity exercise.ab,ti.	2761
35	Exercise based rehabilitation.ab,ti.	316
36	14 or 15 or 16 or 17 or 18 or 19 or 20 or 21 or 22 or 23 or 24 or 25 or 26 or 27 or 28 or 29 or 30 or 31 or 32 or 33 or 34 or 35	403398
37	VO2.ab,ti.	24980
38	Maximum oxygen consumption.ab,ti.	1427
39	VO2max.ab,ti.	12172
40	Peak oxygen consumption.ab,ti.	4824
41	VO2peak.ab,ti.	5937
42	Quality of life.ab,ti.	552010
43	QOL.ab,ti.	92287
44	Health related quality of life.ab,ti.	79548
45	HRQOL.ab,ti.	34553
46	Hospital admissions.ab,ti.	34016
47	Hospitalisation.ab,ti.	32579
48	Hospitalization.ab,ti.	262621
49	Rehospitalisation.ab,ti.	1327
50	Rehospitalization.ab,ti.	8842
51	Fatigue.ab,ti.	186179
52	Muscle strength.ab,ti.	38324
53	Muscular strength.ab,ti.	5218
54	Strength.ab,ti.	400647
55	Feasibility.ab,ti.	317420
56	Aerobic capacity.ab,ti.	7569
57	Pain.ab,ti.	1077406
58	Flexibility.ab,ti.	101481
59	Balance.ab,ti.	317899

C2 General

60 Gait impairments.ab,ti. 698
61 Functional mobility.ab,ti. 3180
62 Body composition.ab,ti. 58668
63 Body mass index.ab,ti. 325400
64 BMI.ab,ti. 381198
65 Body fat.ab,ti. 48883
66 Motor performance.ab,ti. 11406
67 Activity energy expenditure.ab,ti. 922
68 Level of physical activity.ab,ti. 6325
69 Adherence.ab,ti. 222179
70 Immune function.ab,ti. 31639
71 Immune system.ab,ti. 176983
72 Immune parameters.ab,ti. 4792
73 Inflammation markers.ab,ti. 547
74 Inflammatory markers.ab,ti. 45390
75 Cardiorespiratory fitness.ab,ti. 8398
76 Physical fitness.ab,ti. 13831
77 Physical activity.ab,ti. 18206
78 Cardiovascular function.ab,ti. 9539
79 Cardiovascular structure.ab,ti. 54
80 Range of motion.ab,ti. 50904
81 Bone mineral density.ab,ti. 66258
82 Coordination.ab,ti. 132369
83 Risk of infection.ab,ti. 27313
84 Exercise capacity.ab,ti. 24676
85 Endurance.ab,ti. 43790
86 Sleep.ab,ti. 296947
87 Motor function.ab,ti. 38987
88 Ankle dorsiflexion.ab,ti. 3320
89 General health domain.ab,ti. 138
90 Psychosocial health.ab,ti. 2883
91 Functional capacity.ab,ti. 23699
92 Body structure.ab,ti. 1259
93 Body function.ab,ti. 1862
94 Activity.ab,ti. 3531423
95 Participation.ab,ti. 222961
96 Freedom.ab,ti. 67600
97 Adverse effects.ab,ti. 212082
98 37 or 38 or 39 or 40 or 41 or 42 or 43 or 44 or 45 or 46 or 47 or 48 or 49 or 50 or 51 or 52 or 53 or 54 or 55 or 56 or 57 or 58 or 59 or 60 or 61 or 62 or 63 or 64 or 65 or 66 or 67 or 68 or 69 or 70 or 71 or 72 or 73 or 74 or 75 or 76 or 77 or 78 or 79 or 80 or 81 or 82 or 83 or 84 or 85 or 86 or 87 or 88 or 89 or 90 or 91 or 92 or 93 or 94 or 95 or 96 or 97 7761485
99 Systematic review.ab,ti. 297356
100 13 and 36 and 98 and 99 773
101 limit 100 to human 759

C2 General

Table S1. Overall risk of bias of original studies.

**Wolin et al., 2010 [24]**	▪ 10/14 studies → high quality (quality assessment list with eight items)
**Chang et al., 2013 [16]**	▪ Acceptable overall methodological quality (standardized critical appraisal instruments (Appendix I) from the Joanna Briggs Institute Meta Analysis of Statistics Assessment and Review Instrument)
**Grimshaw et al., 2016 [6]**	▪ High risk of bias (Downs and Black Checklist and The Critical Review Form–Qualitative Studies)<br>▪ Poor overall methodological quality
**Braam et al., 2016 [5]**	▪ High risk of bias (definitions as described in the Cochrane Handbook for Systematic Reviews of Interventions)<br>▪ Moderate to very low overall quality of the evidence (GRADE)
**Bhardwaj et al., 2017 [15]**	▪ Overall low-quality grading of included studies? (self-developed quality assessment tool)
**Mizrahi et al., 2017 [20]**	▪ 6/13 studies → weak overall methodological quality<br>▪ 4/13 → moderate<br>▪ 3/13 → strong<br>(Effective Public Health Practice Project Quality Assessment Tool for Quantitative Studies (EPHPP))
**Morales, et al., 2020 [21]**	▪ Low overall methodological quality (PEDro Scale)
**Santos et al., 2020 [22]**	▪ Regular overall methodological quality (PEDro scale based on the Delphi method, mean score = 5.6 points, range 4-9)
**Coombs et al., 2022 [17]**	▪ Risk of bias: moderate for 8 RCTs and 1 CCT + high for 3 RCTs and 1 CCT (revised Cochrane risk-of-bias tool for randomized trials (ROB 2) or risk of bias in non-randomized studies of interventions (ROBINSI))
**Martha et al., 2020 [19]**	▪ High risk of bias (Cochrane Collaboration tool and ROBINS-I tool)<br>▪ Overall quality of the evidence: low and very low (determined by the GRADE system)
**Morales et al., 2020 [9]**	▪ Fair overall quality of included studies (PEDro, mean score = 4, range 0–8)
**Khaleqi-Sohi et al., 2021 [18]**	▪ Moderate overall methodological quality (PEDro, men = 6, range 5-7)
**Shi et al., 2022 [23]**	▪ Moderate risk of bias (Cochrane Handbook for Systematic Reviews of Interventions)

C2 General
